# Prediction and realisation of high mobility and degenerate p-type conductivity in CaCuP thin films†

**DOI:** 10.1039/d2sc01538b

**Published:** 2022-04-26

**Authors:** Joe Willis, Ivona Bravić, Rekha R. Schnepf, Karen N. Heinselman, Bartomeu Monserrat, Thomas Unold, Andriy Zakutayev, David O. Scanlon, Andrea Crovetto

**Affiliations:** Department of Chemistry, University College London 20 Gordon Street London WC1H 0AJ UK d.scanlon@ucl.ac.uk; Thomas Young Centre, University College London Gower Street London WC1E 6BT UK; Diamond Light Source Ltd, Harwell Science and Innovation Campus Didcot Oxfordshire OX11 0DE UK; Theory of Condensed Matter Group, Cavendish Laboratory, University of Cambridge J. J. Thomson Avenue Cambridge CB3 0HE UK; National Renewable Energy Laboratory Golden Colorado 80401 USA andriy.zakutayev@nrel.gov ancro@dtu.dk; Department of Physics, Colorado School of Mines Golden Colorado 80401 USA; Department of Materials Science and Metallurgy, University of Cambridge 27 Charles Babbage Road Cambridge CB3 0FS UK; Department of Structure and Dynamics of Energy Materials, Helmholtz-Zentrum Berlin für Materialien und Energie, GmbH Berlin Germany

## Abstract

Phosphides are interesting candidates for hole transport materials and p-type transparent conducting applications, capable of achieving greater valence band dispersion than their oxide counterparts due to the higher lying energy and increased size of the P 3p orbital. After computational identification of the indirect-gap semiconductor CaCuP as a promising candidate, we now report reactive sputter deposition of phase-pure p-type CaCuP thin films. Their intrinsic hole concentration and hole mobility exceed 1 × 10^20^ cm^−3^ and 35 cm^2^ V^−1^ s^−1^ at room temperature, respectively. Transport calculations indicate potential for even higher mobilities. Copper vacancies are identified as the main source of conductivity, displaying markedly different behaviour compared to typical p-type transparent conductors, leading to improved electronic properties. The optical transparency of CaCuP films is lower than expected from first principles calculations of phonon-mediated indirect transitions. This discrepancy could be partly attributed to crystalline imperfections within the films, increasing the strength of indirect transitions. We determine the transparent conductor figure of merit of CaCuP films as a function of composition, revealing links between stoichiometry, crystalline quality, and opto-electronic properties. These findings provide a promising initial assessment of the viability of CaCuP as a p-type transparent contact.

## Abstract 2

We synthesize air-stable, p-type CaCuP thin films with high hole concentration and high hole mobility as potential p-type transparent conductors. We study their optoelectronic properties in detail by advanced experimental and computational methods.

## Graphical abstract

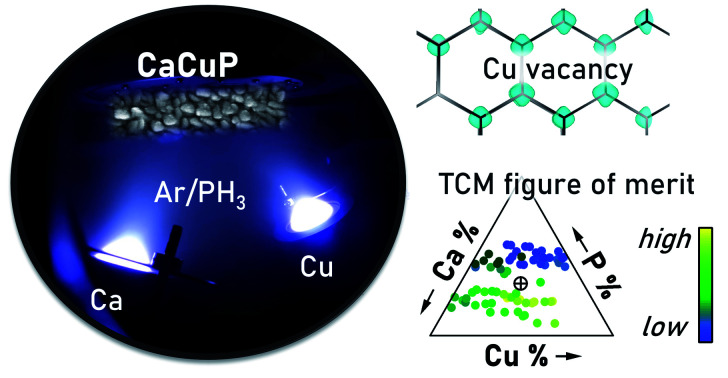

## 1. Introduction

High performance p-type transparent conducting materials (TCMs) have eluded researchers for decades. As we sit on the cusp of a new era of optoelectronic technologies, such as super high-resolution OLED displays (for which p-type TCMs are preferred over n-type electrodes)^1^ and even fully transparent display screens and electronics,^2^ the desire and necessity for reliable p-type TCMs is increasing inexorably. This has led to an explosion in new avenues of p-type TCM research,^3–6^ with database-screening and machine-learning driven discovery bringing countless potential materials to the attention of the community.^7–12^ Non-oxides are particularly attractive candidates as p-type TCMs because the valence orbitals on the anion tend to be more diffuse than the oxygen 2p orbitals at the heart of more traditional materials, and often form valence band maxima with greater dispersion. This trend was quantified by Varley and co-workers in 2017, where they surveyed 30000 structures on the Materials Project,^13^ finding that phosphides had a median hole effective mass around 5*m*_e_ lower than that of oxides.^14^ This is quite a significant difference, suggesting that phosphides, and indeed other non-oxides, are an exciting class of materials for high hole mobility applications.

In addition to good valence band dispersion, an optical band gap exceeding 3.1 eV is required for transparent conductors, corresponding to the shortest wavelength of visible photons. Varley and co-workers also compared the trends in band gap across oxides, sulfides, nitrides and phosphides, finding that the median empirically corrected fundamental density functional theory (DFT) band gap of the phosphides investigated was around 2 eV, roughly half that of the oxides.^14^ This presents a challenge for phosphides, and in general for non-oxide transparent conductors. However, achieving transparency in materials with a small fundamental band gap is possible. SnO is a bi-polar TCM with an indirect fundamental gap of 0.7 eV, but larger direct gap of 2.7 eV corresponding to the optical transitions, yielding semi-transparency in the visible region.^15^

Similarly, boron phosphide has an indirect gap of 1.8 eV but a much larger direct optical gap of 4.0 eV.^14^ The strong overlap of B 2p and P 3p orbitals at the valence band maximum (VBM) yields a hole effective mass of around 0.3*m*_e_,^14^ and predicted hole mobility is in excess of 900 cm^2^ V^−1^ s^−1^.^16^ This exceptional mobility is attributed to the low effective mass and very weak scattering by polar phonons, due to the low ionicity of BP. This is another advantage of non-oxide materials in general for p-type transparent conducting applications – bonding tends to be more covalent, generally leading to a decreased contribution of polar optical phonon scattering to the total scattering process. There are conflicting reports on the optical transparency of BP, as it seems to strongly depend on synthesis conditions, crystallinity, and epitaxial relationships with the substrate.^17,18^

A current state-of-the-art p-type transparent conducting material (TCM) is copper iodide (CuI), with a figure of merit surpassing that of many oxide based p-type TCMs.^19^ CuI possesses a direct band gap of around 3.1 eV, and has a doubly degenerate VBM with average hole effective masses of 0.3*m*_e_ and 2.1*m*_e_ for light and heavy holes respectively, indicating relatively good band dispersion.^20^ The copper vacancy is predicted to be the main source of conductivity under I-rich growth conditions, where it is not charge compensated by native n-type defects.^20,21^ Room temperature reactive sputter deposition followed by heating in an iodine atmosphere led to a conductivity of 280 S cm^−1^ while retaining up to 85% optical transmission.^19^ Thin films of CuI have been trialled successfully as thin film transistors,^22^ while single crystals of CuI have recently been grown for the first time, capable of acting as a hybrid blue LED.^23^ The facile deposition, wide direct band gap, self-doping mechanism (under I-rich conditions) and absence of secondary phases make CuI a very attractive p-type transparent conducting non-oxide.

**Fig. 1:**
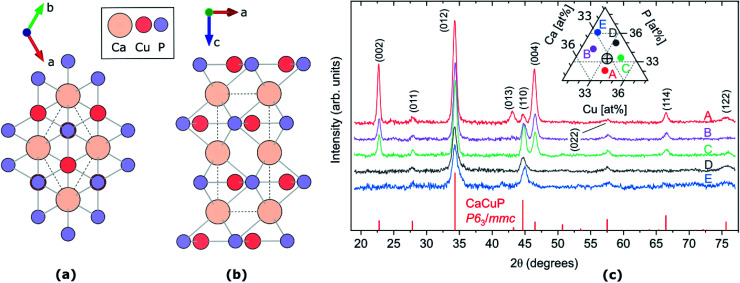
(a and b) Crystal structure of CaCuP, space group *P*6_3_/*mmc*, along *c*-axis (a) and *b*-axis (b). The dotted line represents the primitive unit cell, Ca, Cu and P atoms are represented by pale orange, dark orange and lilac circles, respectively. Visualised using VESTA.^27^ (c) XRD patterns of five CaCuP films with different compositions around the stoichiometric point (Ca : Cu : P = 1 : 1 : 1). The five compositions are shown in the ternary diagram in the inset, where the stoichiometric point is shown as a cross-hair. The diffraction line intensities expected for randomly oriented CaCuP in the *P*6_3_/*mmc* structure^26^ are shown at the bottom.

Combining the Cu 3d and P 3p valence orbitals is another viable technique for achieving high valence band dispersion, and was investigated in the ternary phosphides MCuP, where M = Mg, Ca, Ba, and Sr, by Williamson *et al.* in 2017.^24^ MgCuP crystallises in the *Pnma* space group,^25^ resulting in a Cu atom tetrahedrally coordinated to four P atoms, while CaCuP, BaCuP and SrCuP crystallise in the *P*6_3_/*mmc* space group,^25,26^ where the Cu adopts a trigonal planar configuration with the P atoms forming hexagonal layers of Cu–P sheets (Fig. 1a and b). The trigonal planar coordination of Cu allows for greater spatial overlap and mixing of the Cu 3d states and P 3p states at the valence band maximum compared to tetrahedrally coordinated Cu in MgCuP, at the cost of more anisotropic behaviour. Of the three *P*6_3_/*mmc* structures studied computationally, CaCuP emerged as the most promising candidate for p-type TCM applications, and when synthesised as a nominally undoped powder sample it displayed p-type conductivity of around 500 S cm^−1^ (±50 S cm^−1^). This is extremely high for a pressed pellet subject to grain boundary and interface scattering. These samples also showed a strong optical absorption onset at around 2.7 eV, in good agreement with predicted behaviour.^24^ However, the optical absorption coefficient of a powder sample is not easily accessible. These preliminary results justify a more systematic exploration of thin-film CaCuP to investigate potential deposition methods, optical properties, the origin of its electrical conductivity, and the dependence of the latter on growth conditions.

In this work, we find that polycrystalline CaCuP thin films with degenerate p-type conductivity and high mobility can be deposited by reactive sputtering. The electrical properties are sensitive to the crystalline quality of the film, which decreases as a function of increasing P content. The optical properties are also sensitive to the crystallinity, with stronger optical absorption recorded in the films of lower crystalline quality. We use density functional theory (DFT) to investigate the defect chemistry of CaCuP, finding the copper vacancy to be extremely low in energy and stable into the valence band maximum, giving rise to degenerate semiconducting behaviour. Charge transport theory is used to model the electrical properties of CaCuP, in reasonable agreement with experimental data, and confirm the trend of improved electrical behaviour with crystal quality. We model the absorption behaviour including phonon-mediated indirect transitions, and find that the experimental absorption coefficient is significantly higher than the theoretical prediction. We calculate the band alignment and TCM figure of merit for CaCuP, and compare these to other p-type transparent conductors.

## 2. Methodology

### 2.1. Experimental methods

CaCuP films were deposited on Corning Eagle XG borosilicate glass and crystalline silicon substrates placed in symmetry-equivalent positions with respect to the deposition sources. The deposition technique was reactive radio-frequency (RF) magnetron co-sputtering of metallic Ca and Cu targets in an Ar/PH_3_ atmosphere. The total sputter pressure and substrate platen temperature were fixed at 5 mTorr and 490 °C respectively in all depositions. Unless otherwise stated, the films presented in this article have thickness between 200 nm and 300 nm. Most characterization was conducted with mapping-type measurements and the resulting combinatorial characterisation data was managed with the custom COMBIgor suite.^28^ The films on Si were employed for Rutherford backscattering, X-ray fluorescence, and ellipsometry measurements. The films on glass were employed for X-ray diffraction, optical transmission and reflection, and electrical measurements. Consistency between the films grown on the two types of substrates is discussed in the ESI.†

X-ray diffraction (XRD) measurements were conducted using Cu K_α_ radiation and a 2D detector. Elemental composition and film thickness were determined by X-ray fluorescence (XRF) calibrated by Rutherford backscattering spectrometry (RBS) and spectroscopic ellipsometry. Sheet resistance was measured in the substrate plane with a collinear four-point probe. Temperature-dependent Hall carrier concentration and mobility were measured in the substrate plane in the van der Pauw configuration. The absorption coefficient of a stoichiometric film was determined by measuring transmission *T* at normal incidence and reflection *R* at near-normal incidence using an integrating sphere to include the diffuse component of both transmission and reflection. The absorption coefficient *α* was extracted by a standard relationship (see ESI†). The absorption coefficients of a larger number of samples were obtained using only direct transmission and specular reflection (both at normal incidence) measured in a custom-built setup with mapping capabilities. The optical functions of CaCuP (refractive index and extinction coefficient) extending into the UV range were measured by spectroscopic ellipsometry on a thinner film to minimise scattering and depolarisation effects^29,30^ Further details on the experimental methods can be found in the ESI.†

### 2.2. Computational methods

First-principles calculations were performed using DFT^31,32^ within the projector augmented-wave method (PAW)^33,34^ as implemented in the code VASP.^35–38^ We used a plane-wave energy cut-off of 400 eV, and a *Γ*-centred 7 × 7 × 4 *k*-point mesh to describe the Brillouin zone of the primitive cell. The electrons considered as valence for each element are as follows: Ca 3p^6^4s^2^ (8 total), Cu 3d^10^4s^1^ (11 total), P 3s^2^3p^3^ (5 total). We use the PBEsol functional^39^ for calculating the vibrational properties and the hybrid PBE0 functional^40,41^ for all other calculations, in accordance with previous simulations on CaCuP.^24^ Defect formation energies were calculated according to the Lany–Zunger correction scheme,^42–45^ thermodynamic stability analysis computed with the CPLAP software,^46^ charge transport properties calculated using the AMSET code,^47^ absorption calculations performed using the independent particle approximation (IPA), the electric dipole approximation^48^ and the Williams–Lax theory,^49–51^ and the surface band alignment calculations were performed using the SURFAXE code.^52^ Further details on the computational methods can be found in the ESI.†

## 3. Results and discussion

### 3.1. Crystal structure and stability

The CaCuP films deposited by reactive sputtering at 490 °C are polycrystalline. In Fig. 1c we show the XRD pattern of five films around the stoichiometric point to illustrate the main trends in their structural properties as a function of the composition. In each film, the diffraction peaks above the noise level can be attributed to CaCuP in the *P*6_3_/*mmc* structure.^26^ Changes in the Cu/Ca ratio do not have a strong influence on the XRD pattern. On the other hand, substantially different patterns are obtained at different P contents (moving vertically in the ternary diagram in the inset of Fig. 1c). When the P content is more than ∼2% above the ideal stoichiometry (Films D and E) the peaks become broader and less intense, indicating a smaller crystallite size and a higher fraction of non-crystalline material in the film, respectively. Furthermore, the (002) and (004) peaks become less intense for increasing P content, compared to the other peaks in the pattern. This indicates a tendency for the *c*-axis to align closer to the growth direction under P-poor conditions. The grain size of Film C in the substrate plane is on the order of 50 nm as estimated by SEM images (Fig. S9†).

**Fig. 2:**
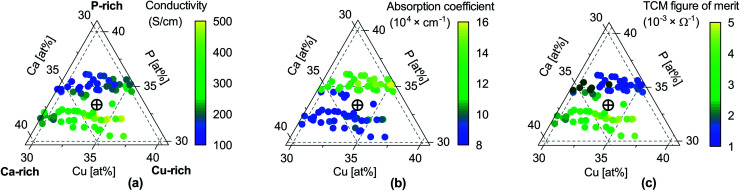
Properties of CaCuP films as a function of composition. (a) Electrical conductivity *σ*; (b) absorption coefficient *α* at 550 nm wavelength (centre of the visible region); (c) TCM figure of merit *Φ* = σ/α, expressing the trade-off between conductivity and transparency following Gordon.^53^ Unlike the original Gordon figure of merit, we consider *α* at a single wavelength because the optical transmission of CaCuP films is too low to determine *α* near the UV edge (see Fig. S14†). In each sub-figure, the cross-hair marks the stoichiometric point (Ca : Cu : P = 1 : 1 : 1).

We did not observe degradation in CaCuP films in the composition range displayed in Fig. 2 when storing them in a N_2_-purged box for a few months and occasionally taking them out for measurements under ambient conditions.

The crystal structure of CaCuP is shown in Fig. 1a and b, and is well reproduced by DFT. It consists of trigonal planar Cu atoms (dark orange) coordinated to P atoms (lilac) in the *ab*-plane, with Ca atoms (pale orange) sitting above the hexagonal voids in the c-direction. Table S1† shows the lattice parameters obtained after structure optimisation using various exchange–correlation functionals, as well as a comparison to experiment and previous DFT calculations. An extended discussion of the crystal structure–electronic structure relationship in CaCuP can be found in ref. 24. A phonon dispersion curve is produced from lattice-dynamics calculations using a converged 6 × 6 × 6 *q*-point grid in Fig. S1.† The absence of imaginary phonon modes indicates that CaCuP is dynamically stable.

### 3.2. Electrical properties

#### 3.2.1. Experiment

CaCuP films close to the stoichiometric point have p-type conductivity, as verified by the sign of both the Hall and Seebeck coefficients (Fig. S10 and S11†). The exact value of the conductivity depends on the elemental composition (Fig. 2a). The first trend is a conductivity increase for increasing Cu/Ca ratios (left to right in Fig. 2a) regardless of P content. This is in agreement with the thermodynamic stability calculations (see Fig. 5a) that predict the highest p-type dopability under Ca-poor conditions, suggesting CaCuP can tolerate significant Ca under-stoichiometry. The second trend is a conductivity drop when P/(Cu + Ca) > 1 (moving upwards in Fig. 2a), *i.e.* under P-rich growth conditions. This is in disagreement with the thermodynamic stability calculations, which predict that significant P over-stoichiometry (combined with metal under-stoichiometry) affords the best p-type growth conditions. However, our experiments show that increasing P content leads to a decline in crystalline quality, which may be at least partially responsible for the drop in conductivity. The trends in average *c*-axis texturing *versus* P content (Fig. 1c) also favour a higher conductivity in P-poor films because our measurement technique probes transport in the plane of the substrate. Specifically, a higher mobility is expected in the substrate plane for increasing *c*-axis texturing because the hole effective mass in CaCuP is around 4 times larger in the *Γ* → *A* direction (Fig. S5,† corresponding to the *c*-axis in real space) compared to other high symmetry directions. This is illustrated in the hole density visualisation, Fig. 6.

**Fig. 3:**
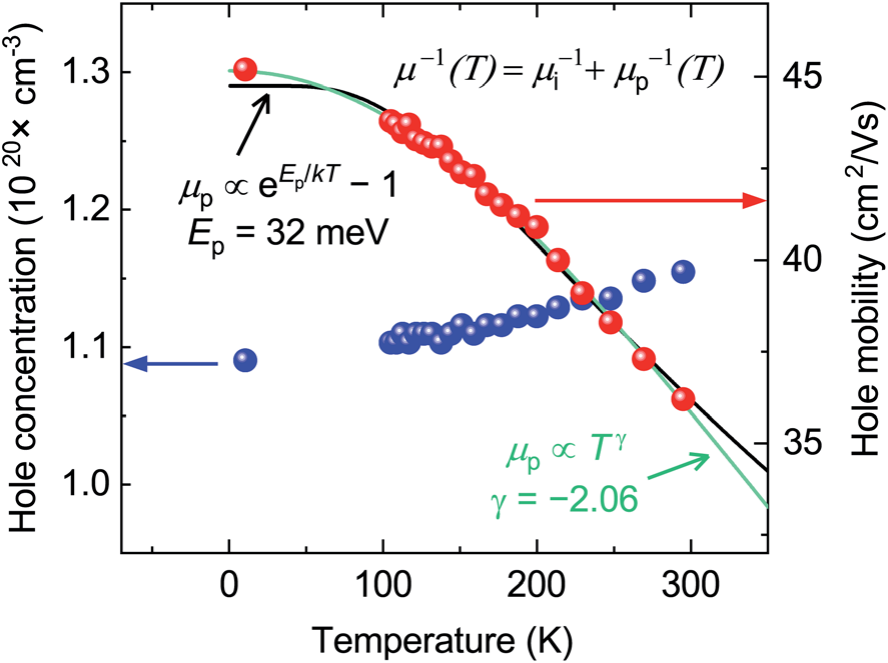
Hall measurements on a near-stoichiometric CaCuP film in the 10 K to 300 K temperature range. Hall hole concentration and mobility plotted in blue and red, respectively. The phonon-limited mobility fit in green is to a generic power law, *μ*_p_(*T*) ≡ *AT*^*γ*^, while the fit in black is to the analytically derived expression for optical-phonon limited mobility, *μ*_p_(*T*) ≡ *B* exp(*E*_p_/*k*_B_*T*) − 1. Fits are explained in greater detail in the text.

A separate film (Ca : Cu : P = 1.03 : 1.09 : 1.00) was deposited in the shape of a Hall cross for further electrical measurements. At room temperature, the (Hall) hole concentration is 1.15 × 10^20^ cm^−3^ and the (Hall) hole mobility is 36.4 cm^2^ V^−1^ s^−1^ (Fig. 3). It is interesting to compare these properties to the case of state-of-the-art TCMs without extrinsic doping. Among p-type materials, CuI is one of the compounds with the highest figures of merit. Its highest reported hole concentration and mobility when deposited as a thin film are 1 × 10^20^ cm^−3^ and 9 cm^2^ V^−1^ s^−1^ respectively,^19^ and 1 × 10^18^ cm^−3^ and 110 cm^2^ V^−1^ s^−1^ when grown as a single crystal.^23^ Among the best n-type TCMs with a long research history (In_2_O_3_ and ZnO), free electron concentrations in non-epitaxial, intrinsically doped films are generally below 4 × 10^20^ cm^−3^ and electron mobilities are below 40 cm^2^ V^−1^ s^−1^.^54,55^ The electrical properties of p-type CaCuP films are therefore on par with the best n-type intrinsic TCMs.

Knowing the Hall carrier concentration and the Seebeck coefficient (Fig. S11†) we can estimate the effective mass of CaCuP using previously developed theory.^56^ This analysis yields an effective mass of 0.44 *m*_e_, in excellent agreement with the direction-averaged heavy hole effective mass (0.43 *m*_e_) obtained from DFT calculations.^24^

The hole concentration of CaCuP films rises very slowly with temperature (Fig. 3). This behavior is typical of a degenerately doped semiconductor,^57^ and is in excellent agreement with defect calculations, which predict degenerate semiconducting behaviour through acceptor levels at or below the valence band maximum, shown in the transition level diagram for the most p-type growth conditions (Fig. 5b). On the other hand, the hole mobility *μ*(*T*) decreases with temperature. This trend can be described by considering two parallel scattering channels, so that μ^−1^(*T*) = μ_i_^−1^ + μ_p_^−1^(*T*). *μ*_i_ is the mobility resulting from ionised impurity scattering (here, the native acceptors). This term is expected to be nearly temperature-independent for a degenerate semiconductor.^58^*μ*_p_(*T*) ≡ *AT*^*γ*^ is a temperature-dependent mobility resulting from phonon scattering. Fitting *μ*_i_, *A* and *γ* to the experimental mobility (shown in green) yields *μ*_i_ = 45.2 cm^2^ V^−1^ s^−1^ and *γ* = −2.06 (Fig. 3). According to theory developed for p-type III–V semiconductors, *γ* = −1.5 for purely acoustic phonon scattering and *γ* < − 1.5 when contributions from optical phonons are also present.^59^ Similar to CaCuP, III–V semiconductors have a doubly degenerate valence band maximum at the *Γ* point and typically *γ* ≃ −2.3.^59,60^

#### 3.2.2. Theory

**Fig. 4:**
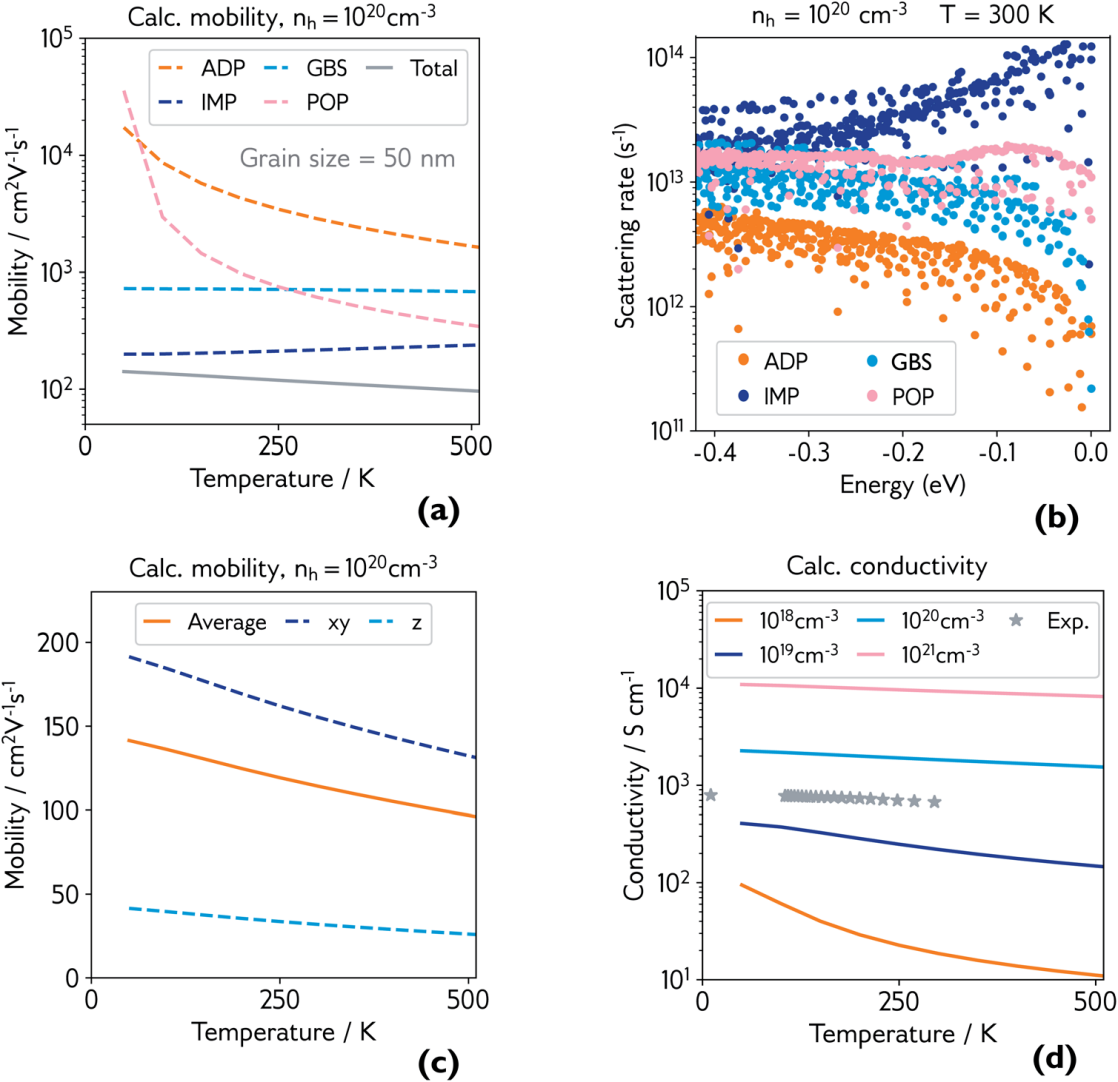
(a) Calculated hole mobility at hole concentration of 1 × 10^20^ cm^−3^ and grain size of 50 nm. Mobility is broken down by individual scattering mechanism (ADP = acoustic deformation potential, IMP = ionised impurities, GBS = grain boundary scattering, POP = polar optical phonons), and the total is shown in grey; (b) explicit scattering rates at hole concentration of 1 × 10^20^ cm^−3^ and room temperature, for a range of energies below the valence band maximum (0.0 eV), broken down by scattering mechanism; (c) calculated directional hole mobility at hole concentration of 1 × 10^20^ cm^−3^; (d) calculated conductivity at various hole concentrations, with experimental conductivity of the film measured in Fig. 3 plotted in grey.

The temperature-dependent electrical properties of CaCuP were also investigated using Boltzmann transport theory (AMSET package)^47^ and are summarised in Fig. 4. Unless otherwise specified, the hole concentration is fixed at *n*_h_ = 1 × 10^20^ cm^−3^ for comparison with experiment. First we consider limits to the hole mobility by the following competing scattering mechanisms: acoustic deformation potentials (ADP), ionised impurities (IMP), grain boundaries (GBS) and polar optical phonons (POP), in Fig. 4a. Note that the grain boundary scattering rate in AMSET is simply set to *v*_g_/*L*, where *v*_g_ is the group velocity and *L* is the grain diameter.^47^ In case other hole-scattering extended defects exist within grains, *L* then represents their average distance. Potential barriers at grain boundaries are ignored, but this may be a reasonable approximation in a material with a high hole concentration.

The dominant mobility-limiting mechanism is ionised impurity scattering, showing a very weak temperature dependence. Grain boundary scattering also has a weak temperature dependence but a lower scattering rate, assuming a grain diameter *L* = 50 nm based on scanning electron microscopy images of the films (Fig. S9†). At around 250 K, polar optical phonon scattering begins to dominate over scattering from grain boundaries, due to its much stronger temperature dependence. Scattering from acoustic phonons has a slightly weaker temperature dependence, and the lowest contribution to the overall scattering rate. The combined effect of these four competing mechanisms is a moderate decrease of the total mobility with temperature, in agreement with experiment. The room-temperature mobility is predicted to be 113 cm^2^ V^−1^ s^−1^, with polar optical phonons becoming increasingly important at high temperatures. These results confirm that it is reasonable to describe the experimental mobility as the reciprocal sum of a temperature independent mobility (representing ionised impurity scattering and potentially grain boundary scattering) and a phonon-limited mobility which is temperature dependent. Since the calculations predict that phonon scattering is predominantly from polar optical phonons, we can replace the generic power law *μ*_p_(*T*) ≡ *AT*^*γ*^ with the analytically derived expression *μ*_p_(*T*) ≡ *B* exp(*E*_p_/*k*_B_*T*) − 1, where *E*_p_ is the energy of the optical phonon mode responsible for hole scattering.^60^ Fitting again the experimental mobility as μ^−1^(*T*) = μ_i_^−1^ + μ_p_^−1^(*T*) with the new expression for *μ*_P_(*T*) yields *E*_p_ = 32 meV (black line, Fig. 3). This value is in good agreement with the effective optical phonon energy of CaCuP (∼ 27 meV) derived from our calculated phonon band structure following ref. 47 (Fig. S1†).

Fig. 4b shows the explicit scattering rates at energies at and just below the valence band maximum. This confirms that ionised impurity scattering is the predominant scattering mechanism in CaCuP at room temperature. Fig. S6† compares the scattering rates at 1 × 10^18^ cm^−3^ and 1 × 10^20^ cm^−3^, where a clear switch in the dominant scattering mechanism can be seen – polar optical phonons dominate at lower hole concentrations, and the mobility has a much stronger temperature dependence. Also shown is the effect of decreasing the grain size to 20 nm, which increases the scattering rate from grain boundaries, lowering the total mobility and therefore conductivity, as observed in experiment. We also present directional mobility data in Fig. 4c. Predicted hole mobility is almost four times greater in the *xy*-direction (in plane) compared to the out of plane (*z*-direction) mobility. This is as expected, considering the layered structure of CaCuP and the high dispersion that is confined to only two directions in the band structure. This anisotropy may explain why the electrical properties of the films with greater *c*-axis texturing are superior, when measured in the plane of the substrate.

Finally, the calculated conductivity at various hole concentrations is plotted in Fig. 4d, with experimental conductivity plotted alongside. The change in dominant scattering mechanism from POP to IMP is accompanied by a weaker temperature dependence of the conductivity with increasing hole concentration.

#### 3.2.3. Discussion

Even though the charge transport calculations roughly reproduce the temperature dependence of the experimental mobility, there is a factor of three difference in room temperature mobility between computation and experiment. We expect this is mostly due to the polycrystalline nature of the films, and possibly due to scattering by non-polar optical phonons, which are not considered in the AMSET workflow. It is plausible that extended intra-grain defects and amorphous inclusions are present in our sputtered films. These crystalline imperfections would increase the GBS term in the calculation, and indeed changing the effective crystallite size to 20 nm (Fig. S6c and d†) drastically changes the contribution of GBS scattering to the mobility, and the room temperature value drops to 90 cm^2^ V^−1^ s^−1^. Additional ionised or neutral defects besides the dominant Cu vacancies are also expected in an off-stoichiometric film. We expect that epitaxial CaCuP films and CaCuP single crystals could display hole mobilities closer to the theoretical limit, as is often observed for many semiconductors.

### 3.3. Defect chemistry

#### 3.3.1. Chemical potential limits

**Fig. 5:**
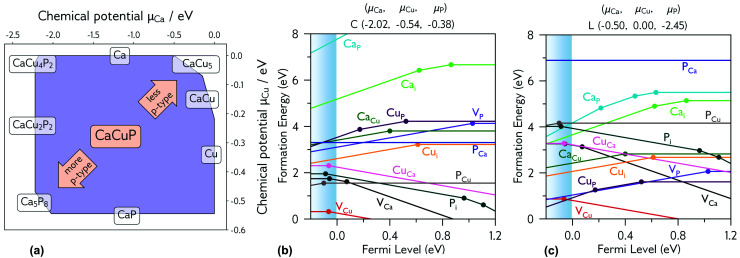
(a) Thermodynamic stability region of CaCuP, calculated using CPLAP;^46^ (b and c) transition level diagrams for CaCuP calculated under (b) strongly p-type and (c) weakly p-type growth conditions. Plotted using AIDE. Fermi level is plotted on the *x*-axis against formation energy on the *y*-axis. The shaded blue region denotes the valence band maximum. Each coloured line denotes a different defect species, and the gradient (positive or negative) denotes the stable charge state at that Fermi level, with horizontal lines denoting neutral charge states. The filled circles denote a thermodynamic transition level (see eqn S(5)†), where two charge states are in equilibrium.

The origin of the p-type conductivity is investigated using hybrid density functional theory. The Materials Project phase diagram (calculated using GGA DFT) of CaCuP was used to identify potential stable limiting phases.^13^ Experimental crystal structures of these phases were relaxed with the PBE0 functional, using a converged *k*-point mesh and the same plane-wave energy cut-off as the CaCuP defect calculations. Fig. 5a shows the thermodynamic stability region of CaCuP, bound by the formation of CaCu, Ca_5_P_8_, CaCu_5_, CaP, CaCu_4_P_2_ and CaCu_2_P_2_. The most p-type growth conditions are predicted to be found at the metal-poor (specifically Ca-poor), phosphorus-rich limit, while the least p-type growth conditions are predicted to occur at the metal-rich, phosphorus-poor limit.

### 3.3.2. Degenerate defect species

**Fig. 6:**
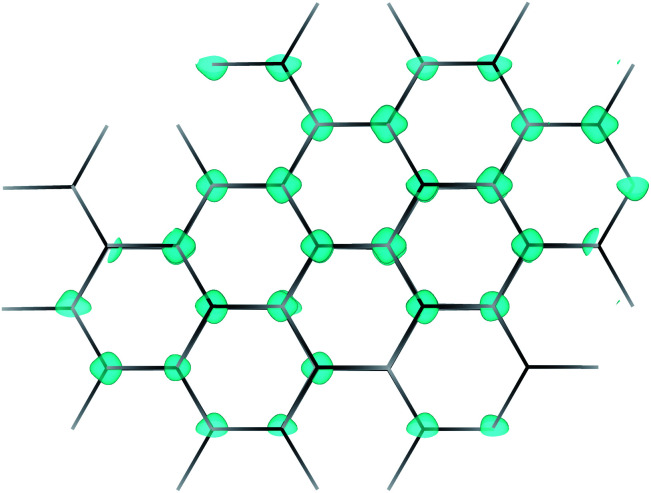
Partial hole density for the copper vacancy in CaCuP. The hole is delocalised throughout the Cu–P plane, in contrast to the largely localised holes that are characteristic of copper vacancies in other p-type transparent conductors.

Fig. 5b and c shows the intrinsic defect transition level diagrams for CaCuP under strongly p-type and weakly p-type growth conditions. The lowest energy defect species under the most p-type growth conditions (Fig. 5b) is the copper vacancy (V_Cu_, red), with a formation energy of 0.32 eV when in the neutral charge state. This species is likely to contribute the majority of charge carriers. It is stable in the −1 charge state (when the defect has accepted an electron, generating a hole in the valence band) until around 0.07 eV below the valence band maximum, indicating degenerate behaviour. This is in agreement with the temperature-independent behaviour of the experimentally measured Hall hole concentration. In fact, there are three other degenerate acceptor defects, namely V_Ca_ (black), P_i_ (interstitial, dark cyan), and Cu_Ca_ (hot pink) which are likely to contribute to p-type conductivity. However, they are not as prevalent as V_Cu_, due to significantly higher formation energies. The P_Cu_ defect (grey) does not charge compensate these three species, as it is only stable in the neutral charge state across all Fermi levels. While the best quality CaCuP films are achieved under Ca-poor conditions, it is not the V_Ca_ defect that is primarily responsible for p-type conductivity. However, the hole density generated by the Ca vacancy in the −1 charge state is still delocalised throughout the Cu–P network (similar to Cu vacancy hole density in Fig. 6), and the disruption to the crystal structure is spatially separated from the conduction pathway. Similar behaviour is observed in La-doping of the n-type transparent conducting perovskite BaSnO_3_. The conduction pathways are comprised of SnO_6_ octahedra, so doping on the Ba site (the A cation site in the ABO_3_ perovskite structure) does not affect the mobility, and instead drives up conductivity by increasing charge carrier concentration.^61–64^ This suggests that cation doping on the Ca site could be an effective strategy for increasing the concentration of charge carriers in CaCuP without significant degradation of the mobility.

We can now consider weakly p-type growth conditions, shown in Fig. 5c. Immediately we notice a difference, with the low energy p-type defects from the previous plot moving up in energy, and n-type defects such as the phosphorus vacancy (V_P_, violet) dropping in energy. However, the lowest energy defect both at the valence band and across all Fermi levels is still V_Cu_, indicating that even under P-poor, metal rich conditions CaCuP is expected to have reasonably good p-type conductivity. Indeed, we see the best conductivity in P-poor films (Fig. 2a).

Clearly the V_Cu_ species is the principal defect in CaCuP, and we can now look at it in greater detail. Fig. 6 shows the partial hole density of V_Cu_. The charge density is delocalised throughout the Cu–P layers in the crystal structure, across the same orbitals that comprise the valence band maximum. Therefore, we expect high hole mobility in two directions, as predicted by the charge transport calculations, and as indicated by the highly disperse bands in the *Γ* → *M* and *Γ* → *K* directions in Fig. S5.† The delocalised nature of this hole is in stark contrast with copper vacancies in p-type transparent conducting oxides. For example, in CuAlO_2_ the hole generated by V_Cu_ is partially localised on the six surrounding Cu atoms, indicative of polaronic behaviour, rather than throughout the whole material.^65^ Similarly, in Cu_2_O, the hole density is localised on the four surrounding O atoms.^66^

#### 3.3.3. Extended defect discussion

It is also briefly worth considering some of the other native defects in CaCuP. The cation interstitials are high in energy, with Ca_i_ significantly less likely to form than Cu_i_. This behaviour can be rationalised from the larger effective ionic radius of Ca^2+^ compared to Cu^+^ (100 pm and 77 pm respectively).^67^ P_i_ is much lower in energy, 1.95 eV in the neutral charge state. This is partly due to its smaller size, but a significant driving force is also the ease of formation of a dimeric P–P species with respect to, *e.g.*, O–O interstitial dimers in metal oxides.^68–72^ The cation on anion antisites, Ca_P_ and Cu_P_, are also high in energy, as significant electrostatic repulsion is generated by substituting a cation onto an anion site that is then surrounded by other cations. Once more, the larger Ca ion causes greater disruption to the local environment, and is therefore the higher energy species. Ca_Cu_ is also a high energy n-type defect, again causing significant lattice distortion, whereas Cu_Ca_ is p-type (effectively self-doping the 2 + site with a 1 + cation), and is separated from the Cu–P planes, resulting in only a small distortion to the local environment. It is worth noting that there is a reasonable extrinsic p-type doping window in CaCuP, and substituting a 1 + cation with a similar ionic radius to Cu^+^ onto the Ca site could result in further generation of holes. Finally, the anion on cation antisites are considered, which are noticeably lower in energy than the cation on anion antisite species. This can be rationalised by noting that P is amphoteric (can act as both a cation and an anion), and so can exist surrounded by P anions by adopting a positive oxidation state. Both the P_Ca_ and P_Cu_ species remain in the neutral charge state (equivalent to P adopting the +5 oxidation state) across all Fermi levels.

### 3.4. Optical properties

#### 3.4.1. Experiment

**Fig. 7:**
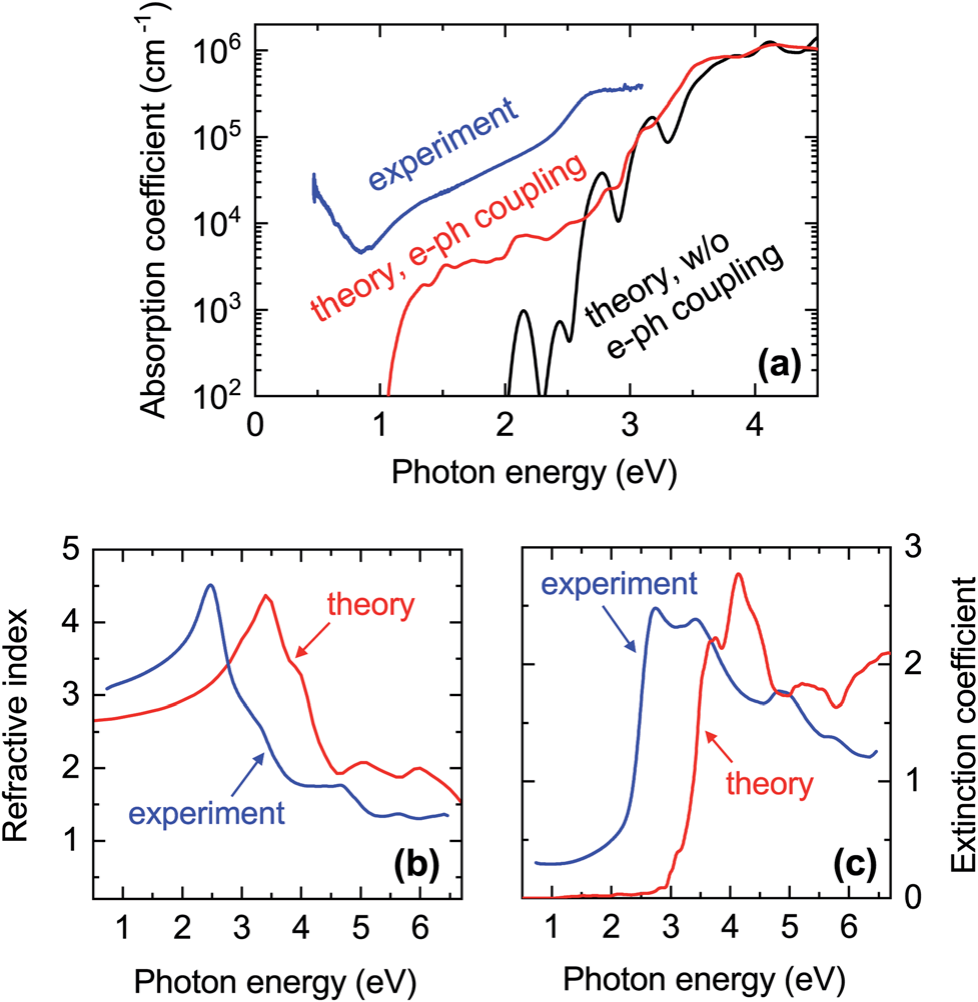
Comparison between experimental and calculated optical properties of CaCuP. (a) Blue line: absorption coefficient extracted from the transmission and reflection measurements (including diffuse components) shown in Fig. S12.† The data is from Film C in Fig. 1. Black line: calculated absorption coefficient with a static lattice and the PBE0 functional (indirect transitions are not included). Red line: calculated absorption coefficient with electron–phonon coupling at 300 K using a 4 × 4 × 4 supercell and the PBE0 functional; (b) refractive index *n* and (c) extinction coefficient *k* extended further into the UV. Blue lines: spectra extracted by spectroscopic ellipsometry on a thinner film. Red lines: calculated spectra with electron–phonon coupling.

The experimentally determined absorption coefficient of a CaCuP film (Film C in Fig. 1c) is plotted in Fig. 7a. The spectrum can be divided into three regions. Absorption below 1.0 eV photon energy can be attributed to free carrier absorption, *i.e.* intra-band absorption of free holes in the valence band. We note that this absorption mechanism is not considered in the calculated coefficient of pristine CaCuP without free carriers. Absorption starts to rise above 1.0 eV photon energy, with indirect transitions from the *Γ* point to between *L* and *M* likely responsible for this (Fig. S5†). Absorption increases more intensely at a second onset around 2.5 eV, which is likely due to direct transitions at the *Γ* point into the conduction band minimum (CBM) and possibly the CBM + 1 (Fig. S5†). This onset is particularly obvious when plotting the extinction coefficient on a linear scale (Fig. 7c). Experimental estimates of the indirect and direct band gaps are 0.9 eV and 2.5 eV respectively (Fig. S12†). The refractive index of CaCuP below the direct band gap is between 3.0 and 3.5, as extracted by ellipsometry (Fig. 7b).

#### 3.4.2. Theory

The computed absorption onset of CaCuP is also shown in Fig. 7a, considering first the static lattice independent particle absorption (IPA). This absorption onset corresponds to the minimum vertical excitation at the optical band gap located at the *Γ* point, with an energy value of 2.17 eV. Even though the direct transition at the *Γ* point is parity-allowed, the absorptivity is rather low. We propose that the second, more intense peak in the static spectrum at 2.71 eV corresponds to a transition at the *Γ* point from the VBM to CBM + 1, with the increased intensity arising from the better overlap of the respective orbitals. Contributions from both of these transitions reasonably yield the experimentally derived direct band gap of around 2.5 eV. Convergence tests with respect to the *k*-point grid suggest that even though the position of the absorption onset is very well converged with a coarse *k*-point grid, the spectral line shape changes significantly and the extremely bright transition at 2.71 eV becomes much less prominent with finer *k*-point sampling (suggesting the very large increase in absorption reported in ref. 24 may be an exaggerated effect of under-convergence).

In Fig. 7a we also show the computed absorption onset at 300 K considering indirect transitions. This simulation includes the effect of electron–phonon coupling, which enables the absorption of a photon across the indirect band gap mediated by the scattering of a phonon. In this case, we see an absorption onset of 1.0 eV, which is significantly closer to the indirect band gap of 1.23 eV at the static lattice value, and in reasonably good agreement with the experimental spectrum. The red-shift of the finite-temperature absorption onset of approximately 200 meV with respect to the static indirect band-gap value can be ascribed to the temperature-dependent renormalisation of the indirect band gap. From this result, we expect the experimental band gap of CaCuP to decrease with increasing temperature as found in most semiconductors. Band gap renormalisations between 50 meV and 200 meV are fairly common when the temperature is elevated from 0 K to 300 K.^73–75^

#### 3.4.3. Discussion

The overall shape of the experimental absorption coefficient is in rather good agreement with the computational results. However, the experimental absorption coefficient is larger than the calculated one by approximately a factor of 10 over the whole spectral range where indirect transitions dominate (Fig. 7a). This discrepancy is particularly detrimental for applications of CaCuP as a transparent conductor, as it leads to very low optical transmission in the visible. By measuring the absorption coefficient as a function of composition (Fig. 2b) we observe that an additional increase in absorption (by around a factor of 2) occurs when moving from slightly P-poor to a slightly P-rich stoichiometry. On the other hand, the dependence of the absorption coefficient on the Cu/Ca ratio is weaker, at least in the vicinity of the stoichiometric point.

To understand these observations, it should be highlighted that the calculated absorption coefficient with electron–phonon coupling assumes a perfectly crystalline material. In a highly disordered material or near grain boundaries, the local lack of crystalline order renders indirect transitions more probable as they can take place without the intervention of a phonon. The grain size of a CaCuP film with stoichiometric P content is only approximately 50 nm (Fig. S9†) and both the crystalline fraction and crystallite size degrade for higher P content. In fact, a strong dependence of optical properties on crystal quality is well known for other indirect band gap materials, such as elemental silicon. Going from fully crystalline to microcrystalline, nanocrystalline and finally fully amorphous Si, the absorption coefficient between the indirect and direct band gap of Si rises accordingly by over an order of magnitude.^76^ Bulk light scattering effects in the measurement or the approximate treatment of excited states by DFT could also contribute to the experiment-theory discrepancy – the anisotropic nature of CaCuP may result in non-negligible excitonic effects which can increase the absorption coefficient, and are not accounted for in regular DFT. Since experimental absorption exceeds computed absorption over a broad spectral range, it is unlikely that an optically active defect can be responsible for it. Nevertheless, we have calculated the absorption and emission profile of the lowest energy defect, V_Cu_, which can be found in Fig. S7.† As expected, defect absorption and emission are closely spaced in energy and are not expected to cause broadband absorption.

#### 3.4.4. TCM figure of merit

In Fig. 2c we plot the ratio between electrical conductivity and absorption coefficient as a function of film composition, to obtain a simple but widely used figure of merit for transparent conductors.^53,77^ In general, slightly P-poor compositions are beneficial for both conductivity and transparency. On the other hand, increasing the Cu/Ca ratio has a beneficial effect on the conductivity but a detrimental effect on the absorption coefficient. Because the former varies over a broader range than the latter, figures of merit are highest (0.005 Ω^−1^) in the Cu-rich region of the diagram. For comparison, the best n-type TCMs have record figures of merit between 1 Ω^−1^ and 10 Ω^−153^ and p-type CuI has reached 0.02 Ω^−1^.^19^ If absorption coefficients closer to our computational predictions could be achieved by, *e.g.*, an improved growth process, the figure of merit of CaCuP could potentially reach some of the highest values known for a p-type TCM.

### 3.5. Band alignment

**Fig. 8:**
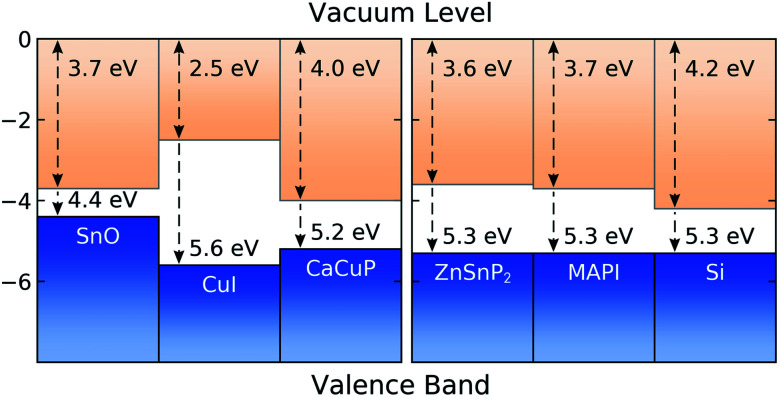
Calculated band alignment of CaCuP, with respect to other state-of-the-art p-type TCMs,^15,83^ displayed to the left, and to some established and emerging semiconductors (experimentally determined), displayed to the right.^80–82^ Numbers at the top of the diagram are electron affinities, and those at the bottom are ionisation potentials. Plotted using BAPT.

In Fig. 8 we show the calculated band alignment of CaCuP, and compare this to other popular p-type transparent conductors (left). The calculations predict an electron affinity of 4.0 eV and ionisation potential of 5.2 eV for CaCuP. P-type semiconductors typically have high-lying valence band maxima and conduction band minima, allowing the facile formation of holes while limiting the formation of n-type defects. This is compared to typical n-type materials, which tend to have a much larger electron affinity which drives occupation of the conduction band by electrons.^78^ The ionisation potential of CaCuP is relatively low, indicating that hole formation is easily achievable – indeed, films of CaCuP display hole (Hall) concentrations on the order of 1 × 10^20^ cm^−3^. In fact, the ionisation potential is lower than that of CuI, which suggests that an overall higher concentration of holes could be achieved with an optimised deposition process.

Ideally, the ionisation potential of a p-type contact material should match the ionisation potential of the semiconductor where holes are to be injected or extracted.^79^ On the right hand side of Fig. 8, we demonstrate that this is the case for CaCuP when compared against the experimentally determined ionisation potentials for two semiconductors of high technological interest (Si and methylammonium lead iodide, MAPI)^80,81^ and an emerging phosphide solar absorber (ZnSnP_2_).^82^ This indicates that CaCuP could integrate well into various types of devices using these materials. In particular, CaCuP could be a convenient hole transport layer in phosphide-based device stacks.

## 4. Conclusions

We demonstrated the feasibility of CaCuP thin film growth by reactive sputtering and evaluated its potential as a p-type transparent conductor. The electrical properties of CaCuP films generally confirm the theoretical expectations: CaCuP is a degenerate p-type semiconductor with a high hole mobility, even in non-epitaxial, polycrystalline films. The Cu–P hexagonal sheets throughout the crystal structure enable delocalisation of the hole generated by low energy copper vacancies, leading to large hole mobility in two directions. Ionised impurity scattering is predicted to be the dominant scattering mechanism at the carrier concentrations achieved experimentally in this study (1 × 10^20^ cm^−3^), and the experimental temperature dependence of the mobility is qualitatively reproduced by the calculations. P content in the films was found to have a strong influence on crystal quality, which in turn determined the opto-electronic properties. Lower crystallinity is correlated with lower electrical conductivity, and also with higher absorption coefficients in the spectral region where indirect transitions dominate. Regardless of film composition, the absorption coefficient of CaCuP films was unexpectedly high. Hence, the maximum figure of merit of CaCuP as a transparent conductor was substantially lower than in the best n-type TCMs and somewhat lower than the highest values achieved by the state-of-the-art p-type TCM CuI.

Nevertheless, band alignment calculations show that the ionisation potential of CaCuP matches the valence band position of various semiconductors of technological interest. Thus, it may be possible to integrate CaCuP as a contact layer into device structures where full transparency is not required. We call on the community for further experimental investigation of CaCuP with focus on its optical properties. Growth of high-quality single crystals would be particularly useful to investigate if the high absorption coefficient of the present CaCuP samples is an intrinsic feature of the material or the consequence of a less-than-ideal growth process. Beyond-DFT calculations that provide an improved description of the band gap (GW theory) and excited states (Bethe–Salpeter formalism) would also provide a clearer picture of the optical properties of CaCuP. Finally, exploration of other thin-film synthesis routes, epitaxial growth, and extrinsic doping would help to build a more robust assessment of CaCuP as a transparent conductor and opto-electronic material in general.

## Author contributions

JW conceptualised and prepared the manuscript, with major contributions from IB and AC, under the guidance of DOS. AC conceptualised and implemented the experimental work, including thin film deposition, most characterisation, and experimental data analysis. Computational work and analysis was performed by JW and IB under the guidance of DOS and BM. RRS and AZ designed and set up the reactive sputtering system. KNH conducted RBS measurements and analysis. Experimental findings were often discussed between AC, TU and AZ. All authors provided input to the manuscript.

## Conflicts of interest

There are no conflicts to declare.

## Data availability

The experimental and computational data shown in this article is available at the following DOI: https://doi.org/10.5281/zenodo.6281750. Additional experimental data is available at the following URL: https://htem.nrel.gov/ (doi: https://doi.org/10.7799/1407128).

## Supplementary Material

SC-013-D2SC01538B-s001
