# Supplementary material for: Prediction and realisation of high mobility and degenerate p-type conductivity in CaCuP thin films
Source: Chem Sci. 2022 Apr 26;13(20):5872–83. doi: 10.1039/d2sc01538b (PMC9132065; doi:10.1039/d2sc01538b)
Supplement: SC-013-D2SC01538B-s001 [file SC-013-D2SC01538B-s001.pdf]

Electronic Supplementary Material (ESI) for Chemical Science.
This journal is © The Royal Society of Chemistry 2022

Prediction and realisation of high mobility and degenerate p-type conductivity in CaCuP thin films: Supplementary Information

Joe Willis,[†,‡,¶] Ivona Bravić,[§] Rekha R. Schnepf,[‖,⊥] Karen N. Heinselman,[‖] Bartomeu Monserrat,[§,#] Thomas Unold,[@] Andriy Zakutayev,[*,‖] David O. Scanlon,[*,†,‡] and Andrea Crovetto[*,‖,@]

†*Department of Chemistry, University College London, 20 Gordon Street, London, WC1H 0AJ, United Kingdom.*

‡*Thomas Young Centre, University College London, Gower Street, London, WC1E 6BT, United Kingdom.*

¶*Diamond Light Source Ltd., Harwell Science and Innovation Campus, Didcot, Oxfordshire, OX11 0DE, United Kingdom.*

§*Theory of Condensed Matter Group, Cavendish Laboratory, University of Cambridge, J. J. Thomson Avenue, Cambridge, CB3 0HE, United Kingdom.*

‖*National Renewable Energy Laboratory, Golden, CO 80401, United States.*

⊥*Department of Physics, Colorado School of Mines, Golden, CO 80401, United States.*

#*Department of Materials Science and Metallurgy, University of Cambridge, 27 Charles Babbage Road, Cambridge, CB3 0FS, United Kingdom.*

@*Department of Structure and Dynamics of Energy Materials, Helmholtz-Zentrum Berlin für Materialien und Energie, GmbH, Berlin, Germany.*

E-mail: andriy.zakutayev@nrel.gov; d.scanlon@ucl.ac.uk; andrea.crovetto@helmholtz-berlin.de

Data availability statement

A broad selection of both experimental and computational data can be accessed in the online repository: https://doi.org/10.5281/zenodo.6281750

Extended experimental methodology

CaCuP films were simultaneously deposited on a $5 \times 5\,\mathrm{cm}^2$ Corning Eagle XG borosilicate glass and a $5 \times 5\,\mathrm{cm}^2$ crystalline silicon substrate. The two substrates were placed in symmetry-equivalent positions with respect to the deposition sources. The deposition technique was reactive radio-frequency (RF) magnetron co-sputtering of metallic Ca and Cu targets (Advanced Engineering Materials, 2″ diameter, 99.9% and 99.99% purity respectively) in an Ar/PH_3 atmosphere. To obtain combinatorial libraries of various material stoichiometries in a single deposition run, the substrate was kept static during the deposition to take advantage of the natural Ca/Cu gradients obtainable by placing the sputter targets on opposite sides of the substrate. Different P contents in CaCuP films were obtained in separate deposition runs by changing the PH_3/Ar flow rate ratio. The total sputter pressure and substrate temperature were fixed at 5 mTorr and 490 °C respectively in all depositions. The temperature was measured at the metallic plate onto which the substrate was clamped during deposition. To obtain a stoichiometric composition in the middle of the substrate, RF powers of 39 W (Ca) and 20 W (Cu) and a PH_3 partial pressure of 0.2 mTorr were necessary. Films of similar compositions generally had similar properties, regardless of the position where this composition was obtained on the substrate. The distance between each target and the middle of the substrate was 16 cm and the deposition rate was about $1.5\,\text{Å}\,\mathrm{s}^{-1}$. Unless otherwise stated, the films presented in this article are 200 nm to 300 nm thick.

Most characterization was conducted with mapping-type measurements and the resulting combinatorial characterisation data was managed with the custom COMBIgor suite.[1] The films on glass were employed for x-ray diffraction (XRD), optical transmission and reflection,

and electrical measurements. For x-ray fluorescence measurements (XRF), it was necessary to use the films on silicon due to the presence of Ca in the glass substrate. Since Rutherford backscattering spectrometry (RBS) and spectroscopic ellipsometry were used to calibrate the XRF spectral analysis routine, CaCuP films on silicon were used for these measurements as well. To ensure compatibility between the data points on Si substrates and the ones on glass substrates, the substrates were placed symmetrically in the deposition chamber and control XRD, XRF, and ellipsometry measurements were conducted on films deposited on both substrates. To ensure that similar temperatures were reached by both substrates, we employed thin glass substrates and carefully contacted them to the heating plate from various sides. By studying the decomposition of Cu_3P films deposited on both substrates at elevated temperatures, we estimate that the temperature difference between the two substrates is less than 20 °C.

XRD measurements were conducted with a Bruker D8 diffractometer using Cu K_α radiation and a 2D detector. To cover the desired 2θ range, two frames were collected with the incidence angle ω fixed at 10° and 22.5°, and the detector centre fixed at 2θ values of 35° and 60°, respectively. The diffraction intensity at each 2θ angle was integrated over the χ range measured by the 2D detector.

Elemental composition and film thickness were determined by XRF in a Bruker Tornado M4 instrument at 15 Torr pressure using a Rh source. XRF spectra were fitted with the Bruker XMethod analysis program. The film composition and thickness extracted by this analysis were calibrated with the composition and thickness of three CaCuP films measured by RBS and spectroscopic ellipsometry, respectively. RBS was performed using a model 3S-MR10 RBS system from National Electrostatics Corporation in a 168° backscattering configuration, using a 2 MeV He^+ beam. Samples were measured until the total integrated charge delivered to the sample was 80 μC, and signals were added together when multiple measurements were taken at a single point. Film composition from RBS was determined by fitting using the RUMP analysis software.[2]

Sheet resistance was measured in the substrate plane with a collinear four-point probe directly contacting the film, and electrical conductivity was derived from it using the XRF-determined thickness. Temperature-dependent Hall carrier concentration and mobility were measured in the substrate plane with a Lake Shore 8425 DC Hall System using the van der Pauw configuration. The CaCuP film employed for this measurement was deposited through a shadow mask to obtain a Hall cross shape, and Ti/Pt contacts were evaporated at the edges of the cross. The DC driving current and magnetic fields were 100 µA and 2 T respectively. The Hall voltage at each temperature was determined as the average of 8 measurements, by reversing sign of the current and of the magnetic field, and by considering two non-equivalent contact geometries.

The absorption coefficient of a stoichiometric film was determined by measuring transmission T at normal incidence and reflection R at near-normal incidence with a Cary 7000 spectrophotometer. The measurement was performed with an integrating sphere to include the diffuse component of both transmission and reflection. The absorption coefficient α was extracted using the relationship

$$\alpha = -\frac{\ln\left(\frac{T}{(1-R)}\right)}{d}, \tag{1}$$

where d is the film thickness as measured by XRF. The absorption coefficients of a larger number of samples were obtained by the same equation using only direct transmission and mirror reflection (both at normal incidence) measured in a custom-built setup with mapping capabilities. Due to the very low transmission of our CaCuP films in the blue part of the visible spectrum, the absorption coefficient is not measured reliably in this region. Therefore, the absorption coefficient at 550 nm wavelength (centre of the visible region) is given as a function of composition in the ternary plots. The optical functions of CaCuP (refractive index and extinction coefficient) extending into the UV range were measured on a thinner film by spectroscopic ellipsometry using a J.A. Woollam M-2000 rotating-compensator ellipsometer and three incidence angles. The optical functions of CaCuP were represented by

Kramers-Kronig-consistent b-spline functions with 0.1 nodes/eV and fitted with the CompleteEase software (J.A. Woollam). As often experienced in ellipsometry measurements,[3,4] thicker CaCuP films often gave unreliable fits with a high standard error, likely due to depolarisation effects linked to surface roughness.

Extended computational methodology

Defect calculations

For defect calculations, we used a roughly cubic 96-atom supercell and a $2 \times 2 \times 2$ Γ-centred $\mathbf{k}$-point mesh. The formation energy of a defect D in a charge state q can be expressed as[5]

$$\Delta E_f^{(D,q)} = \big(E^{(D,q)} - E^H\big) + \sum_i n_i\big(E_i + \mu_i\big) + q\big(\Delta E_F + E^H_{\mathrm{vbm}} + \Delta v^{\mathrm{pot}}\big) + E^{\mathrm{corr}}, \qquad (2)$$

where the first term is the energy difference between a perfect host supercell and defective supercell, the second is a measure of Gibbs free energy change when a number of atoms n_i are added or removed from a system (E_i is the energy per atom, μ_i the chemical potential), the third term is the electron chemical potential when dealing with charged defects (where ΔE_F is the Fermi level of the host supercell, E^H_{vbm} the eigenvalue of the host VBM and Δv^{pot} the potential alignment correction),[5] and the final term contains band-filling[5] and anisotropic image-charge corrections.[6–8] The post-processing corrections used to calculate defect formation energies in this work follow the Lany-Zunger implementation.

The formation energy of CaCuP was calculated from its constituent elements using the equation

$$\Delta E_f^{(\mathrm{CaCuP})} = E^{(\mathrm{CaCuP})} - \big[E^{(\mathrm{Ca})} + E^{(\mathrm{Cu})} + E^{(\mathrm{P})}\big], \qquad (3)$$

for which inequalities of the form

$$\mu_{\mathrm{Ca}} + \mu_{\mathrm{Cu}} + \mu_{\mathrm{P}} < \Delta E_f^{(\mathrm{CaCuP})} \qquad (4)$$

must be true for each stable competing phase, a list of which was obtained from the Materials Project phase diagram for CaCuP.[9] Each species was relaxed using the PBE0 functional and the total energies were used to calculate the chemical potential limits using the CPLAP program.[10] Stability region is shown in the main text, while the CPLAP input file and a data file containing the chemical potential limits can be found in the online repository.

Thermodynamic and optical transition levels are calculated using the equation

$$\epsilon_D(q/q') = \frac{\Delta E_f^{(D,q')} - \Delta E_f^{(D,q)}}{q' - q}. \tag{5}$$

Phonon calculations

For each optimisation run we employ the conjugate gradient algorithm and let the total energy converge within $1\,\mathrm{meV\,atom^{-1}}$. The structural relaxation is stopped when the energy difference is below $10 \times 10^{-10}\,\mathrm{eV}$. The lattice-dynamics calculations use the finite-displacement method[11] in conjunction with non-diagonal supercells[12] to construct the matrix of force constants, which is then Fourier transformed to the dynamical matrix and diagonalised to obtain the vibrational frequencies and eigenvectors. Converged results are obtained with a $6 \times 6 \times 6$ **q**-point grid which is used as a starting point for the Fourier interpolation to a finer grid along high-symmetry lines to construct the phonon dispersion. The phonon-dispersion curve (Figure S1) is calculated using the PBEsol functional.

Absorption calculations

The optical properties of semiconductors and metals such as absorption, reflectance, or energy-loss spectra are all determined by the frequency-dependent complex dielectric function $\varepsilon_1(\omega) + i\varepsilon_2(\omega)$. We evaluate the imaginary part of the dielectric function within the dipole approximation using VASP. For the calculation of the dielectric properties we keep the energy cut-off at $400\,\mathrm{eV}$ and we sample the electronic Brillouin zone using a uniform $14 \times 14 \times 8$ **k**-point grid for the primitive cell and random **k**-point sampling for supercells

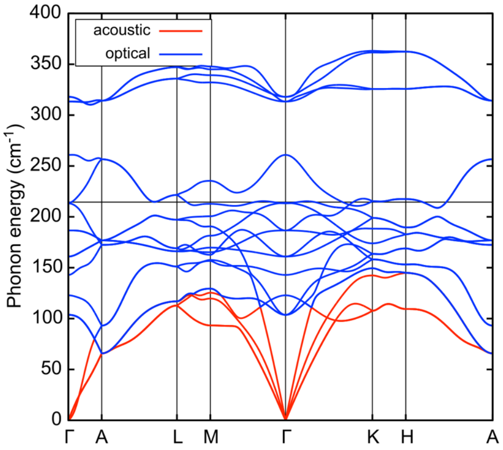

Figure S1: The phonon dispersion curve of CaCuP calculated using a coarse $6 \times 6 \times 6$ $\mathbf{q}$-point grid with the PBEsol functional. The black horizontal line indicates the effective optical phonon energy of CaCuP ($215\,\mathrm{cm}^{-1} \simeq 27\,\mathrm{meV}$) derived from the calculated phonon band structure following Ref.[13]

(convergence tests in Figure S2). Energy conservation in the optical absorption process is enforced by a delta function smeared into a Gaussian function with a width of 80 meV for the static spectrum and 40 meV for the finite temperature spectrum. We report results using the PBE0 functional without spin-orbit coupling. The finite temperature value of ε_2 is determined by:

$$\varepsilon_2(\omega;T) = \frac{1}{\mathcal{Z}} \sum_{\mathbf{s}} \langle \Phi_{\mathbf{s}}(\mathbf{u}) \left| \varepsilon_2(\omega;\mathbf{u}) \right| \Phi_{\mathbf{s}}(\mathbf{u}) \rangle \, e^{\frac{-E_{\mathbf{s}}}{k_{\mathrm{B}}T}}, \tag{6}$$

an approach known as the Williams-Lax theory.[14–16] We subsequently evaluate the corresponding integral using the stochastic Monte-Carlo approximation combined with thermal lines to accelerate the sampling. After calculating ε_2, the real part of the dielectric function ε_1 is obtained using the Kramers-Kronig relation, and the absorption coefficient is $\kappa(\omega) = \omega\varepsilon_2/cn(\omega)$, where c is the speed of light and $n(\omega)$ is the frequency dependent refractive index of the sample. The limit of computational feasibility is reached for a $4 \times 4 \times 4$ supercell in conjunction with random $\mathbf{k}$-point sampling. For a $4 \times 4 \times 4$ supercell (384 atoms) the dielectric function of CaCuP converges using only two sampling points.

We evaluate various convergence parameters for the calculation of the finite temperature absorption coefficient of CaCuP. All calculations are conducted with the PBE0 exchange

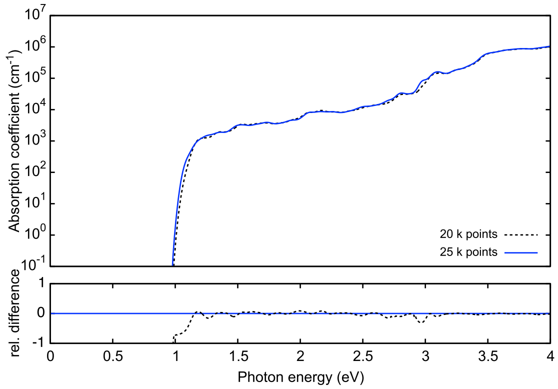

(a)

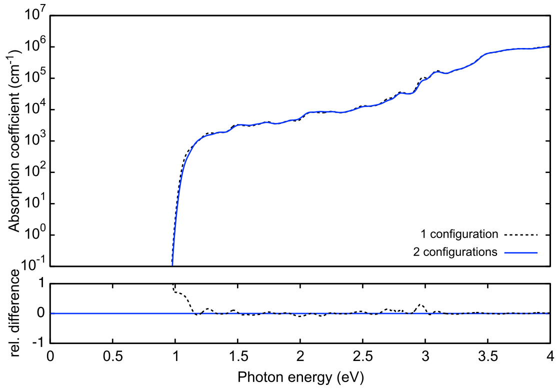

(b)

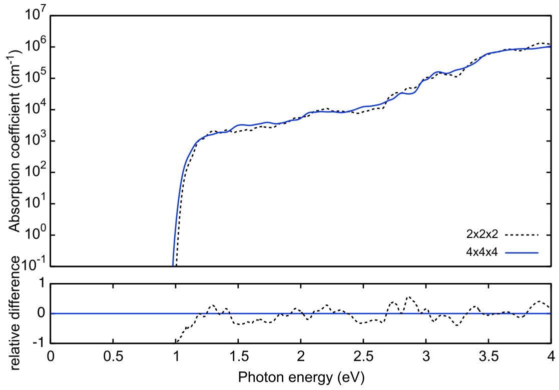

(c)

Figure S2: Convergence tests for absorption calculations. (a) Absorption coefficient of CaCuP at 300 K calculated using a $4 \times 4 \times 4$ supercell. The diagram illustrates the convergence with respect to the number of random electronic **k**-points used to sample the electronic Brillouin zone; (b) Absorption coefficient of CaCuP at 300 K calculated using a $4 \times 4 \times 4$ supercell. The diagram depicts the convergence with respect to the number of thermal line configurations sampled in the thermodynamic average; (c) Absorption coefficient of CaCuP at 300 K calculated using a $2 \times 2 \times 2$ and a $4 \times 4 \times 4$ supercell.

correlation functional using an energy cut-off of 400 eV at 300 K. We first test the convergence with respect to the number of electronic **k**-points used to sample the electronic Brillouin zone and find that 25 **k**-points are sufficient to provide a converged spectrum in proximity to the absorption onset. We then conduct convergence tests on the number of configurations. As already highlighted in prior studies,[17–19] the convergence of the dielectric function is achieved using only very few atomic configurations. In CaCuP we obtain sufficient convergence with only two configurations. Finally, we calculate the absorption spectrum at

300 K using different supercell sizes to evaluate convergence with respect to the **q**-point grid. The largest supercell that is computationally achievable is the $4 \times 4 \times 4$ supercell, corresponding to a $4 \times 4 \times 4$ **q**-point grid. In Figure S2c, we see that the differences between the $2 \times 2 \times 2$ and $4 \times 4 \times 4$ results are small and do not affect the physical interpretation of the results. We finally note that for the calculation of the imaginary part of the dielectric function, energy conservation is imposed by smearing the delta function with a Gaussian. The results reported in the main text correspond to using a smearing width of 40 meV. Tests with a smearing width of 10 meV show that the main conclusions of the manuscript do not change in that smearing range. Overall, the results reported in the main text are obtained using a $4 \times 4 \times 4$ supercell, averaging over two configurations on thermal lines, and including 25 random **k**-points in the Brillouin zone of the supercell (corresponding to 1600 **k**-points in the primitive cell).

Charge transport calculations

Charge transport calculations were performed using the code AMSET,[13] which calculates scattering rates by applying the momentum relaxation time approximation to the Boltzmann transport equation from relatively inexpensive DFT calculations. Scattering mechanisms include acoustic deformation potential scattering (calculated from n- and p-type deformation potentials and the elastic constant of the material), piezoelectric scattering (high-frequency dielectric constant, elastic constant and piezoelectric coefficient – CaCuP was not found to be piezoelectric), ionised impurity scattering (static dielectric constant), and polar electron-phonon scattering (polar optical phonon frequency, from density functional perturbation theory calculations, and both static and high-frequency dielectric constants). Grain boundary scattering is simulated by defining the grain diameter as the mean free path of holes and simply setting the scattering rate as v_g/L, where v_g is the group velocity and L is the grain diameter.[13] All of the parameters (dielectric constants, potentials, wavefunctions, etc.) used to calculate the charge transport properties can be found in the `settings.yaml` file in the

online data repository.

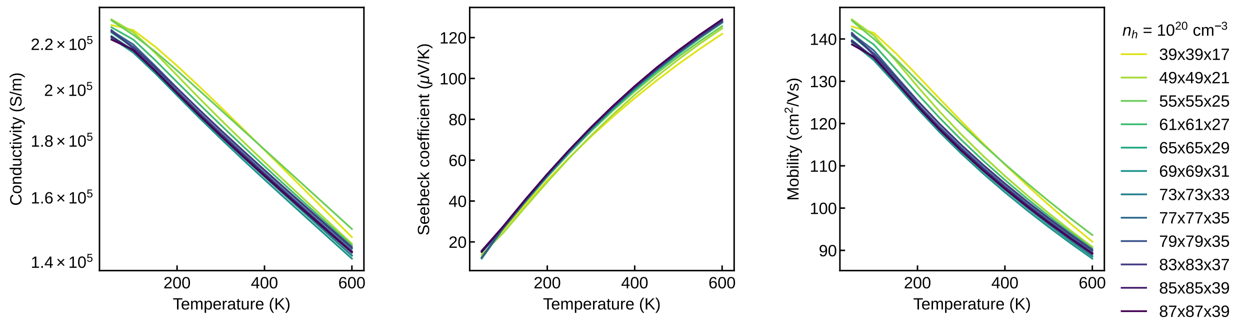

Figure S3: Convergence of various charge transport properties with respect to total **k**-point mesh.

The crystal structure was relaxed using "tight" convergence criteria, with changes in energy below 1×10^{-8} eV for electronic steps and 1×10^{-5} eV for ionic steps required. For the dense uniform **k**-point single-shot calculation, sampling of $14 \times 14 \times 7$ weighted **k**-points plus $17 \times 17 \times 8$ zero-weighted **k**-points was used. For the DFPT calculations, convergence was reached using a plane-wave cut-off of 450 eV and $12 \times 12 \times 6$ **k**-points. The settings file used for the AMSET post-processing is provided in the online repository. Convergence of the charge transport properties with respect to **k**-point density is shown in Figure S3, with a total mesh (using AMSET **k**-points, a combination of **k**-points plus interpolation factor) of $79 \times 79 \times 35$ being used.

Band alignment calculations

The core-level alignment approach was used to align the core state electron eigenvalues to the vacuum level. A slab-gap model (a slab in the centre of the unit cell with a vacuum at each end) was constructed from the PBE0 relaxed bulk structure with a (100) termination, slab thickness of 30 Å and vacuum thickness of 30 Å using the SURFAXE code.[20] The planar average of the electrostatic potential was calculated within SURFAXE and the plateau of this was taken to be the energy of the vacuum. Convergence of the macroscopic electrostatic potential with respect to lattice vector (achieved at a value of 14 Å) is shown in Figure S4.[21–25]

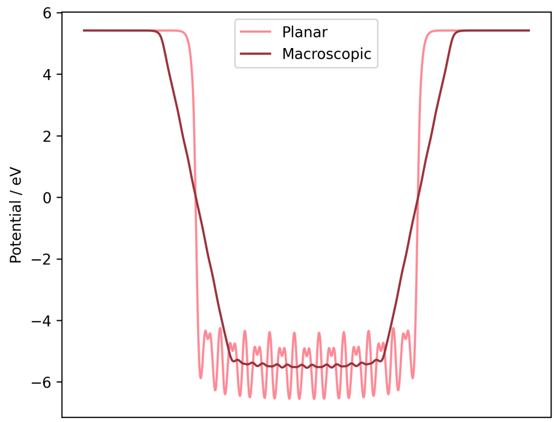

(a) Lattice vector value of 10 Å.

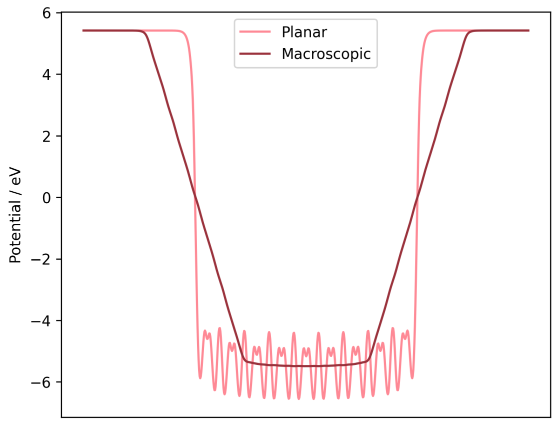

(b) Lattice vector value of 14 Å.

Figure S4: Planar and macroscopic electrostatic potential of CaCuP slabs. Plateau achieved with a value of 14 Å.

Supplementary information

Table 1: Converged lattice parameters a and c calculated using different levels of theory.

Functional	a / Å	c / Å
PBE	4.036	7.939
PBEsol	3.989	7.790
PBE0	4.045	7.810
PBE0[26]	4.05	7.83
Experiment (RT)[27]	4.06	7.80

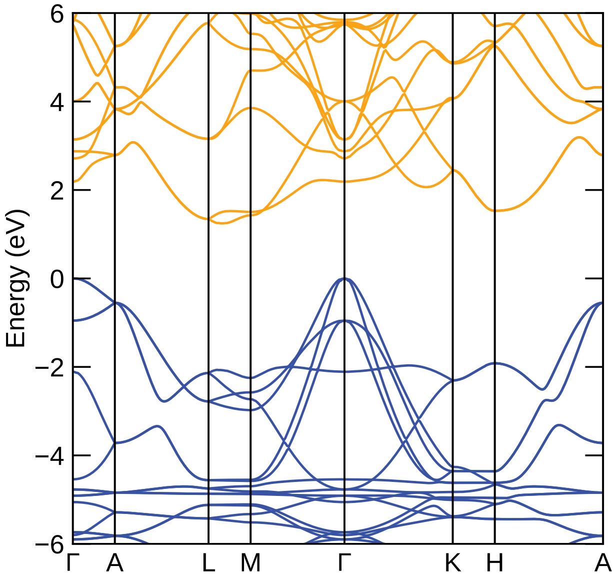

Figure S5: Band structure of CaCuP, direct gap of 2.19 eV, indirect gap of 1.25 eV, plotted using SUMO.[28]

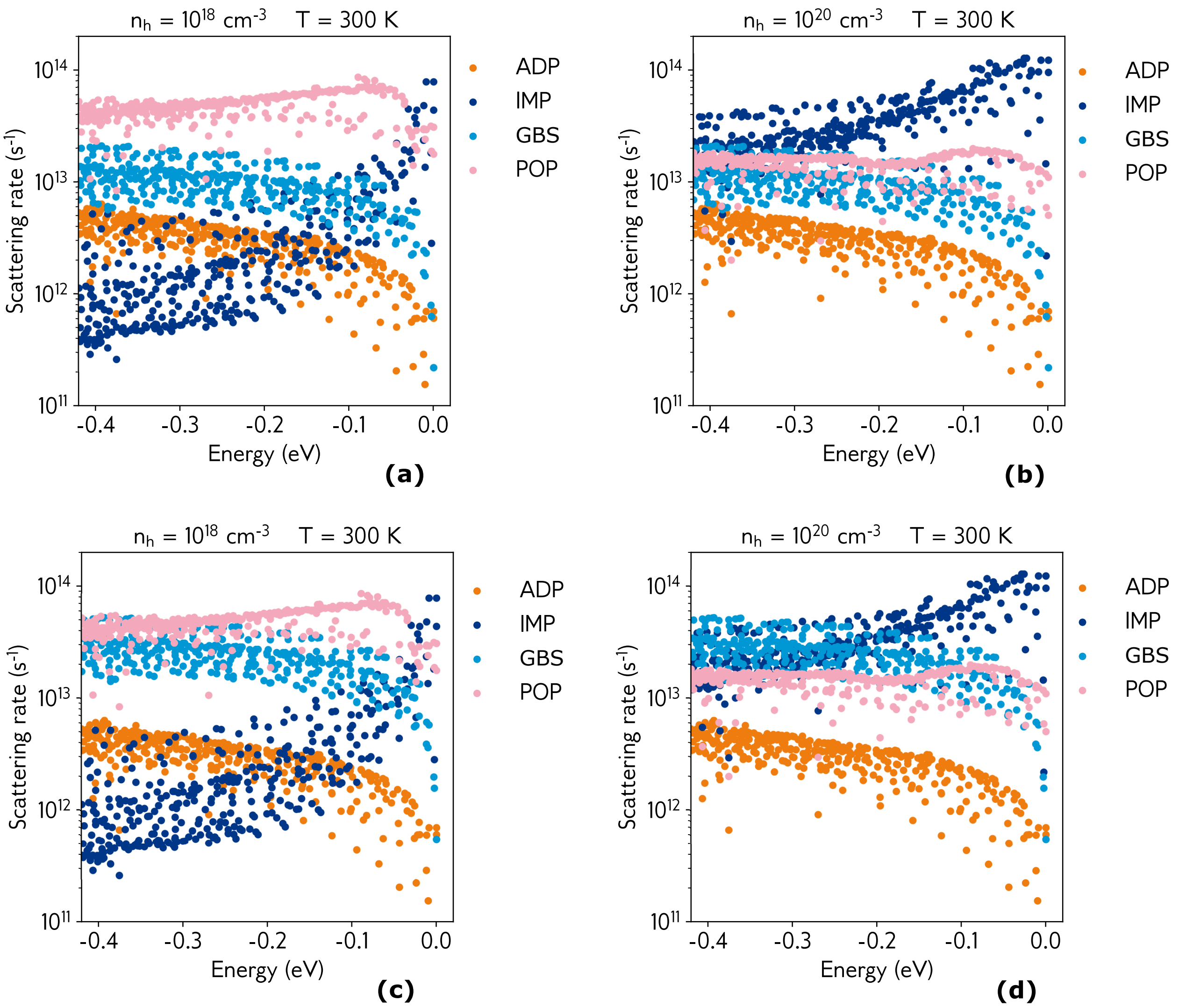

Figure S6: Calculated room temperature scattering rates with a grain boundary size of (a-b) 50 nm and (c-d) 20 nm at low and high hole concentrations. Zero on the energy scale denotes the valence band maximum. At low hole concentrations, POP scattering is rate determining, giving mobility and conductivity a strong temperature dependence, whereas at high carrier concentrations, IMP is rate determining, reducing the temperature dependence. Contribution to the total scattering rate from grain boundary scattering (GBS) increases when grain size is reduced to 20 nm.

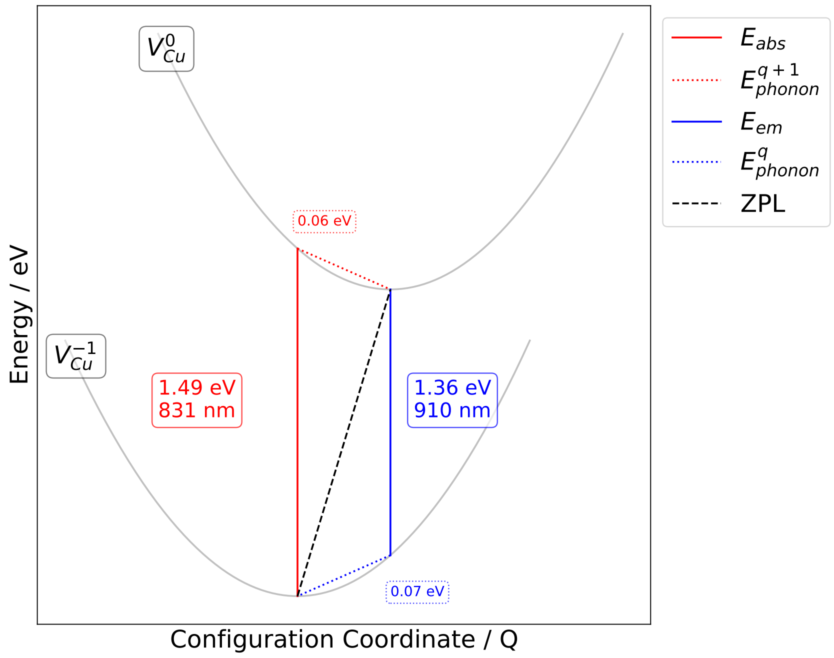

Figure S7: Absorption of an electron to the CBM from the V_{Cu} defect, and emission from the CBM to the defect.

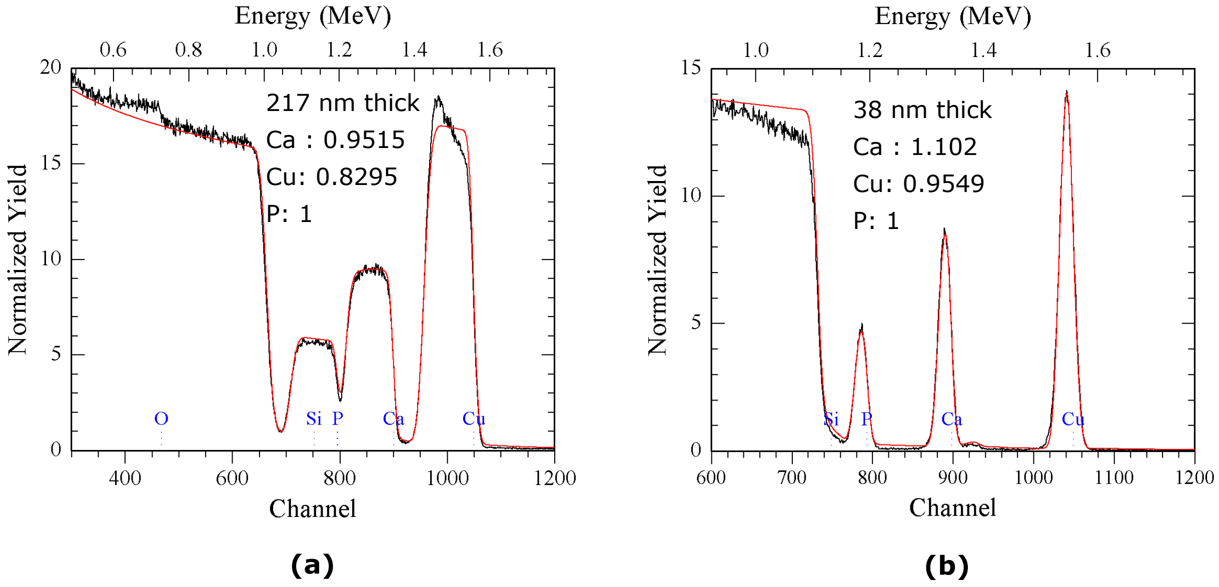

Figure S8: Examples of RBS spectra of CaCuP films used to calibrate high-throughput composition measurements by XRF. For each sample, we show the fitted film thickness (assuming a density of $4.01\,g/cm^3$) and the fitted stoichiometric coefficients of Cu, Ca, and P.

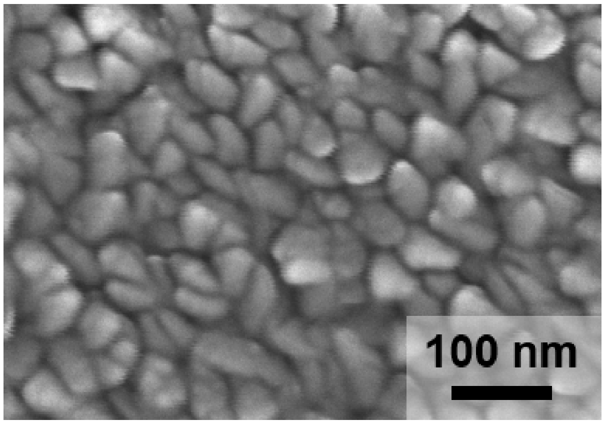

Figure S9: SEM image of a near-stoichiometric CaCuP film using a 5 kV beam voltage and a secondary electron detector. The in-plane grain size is around 50 nm.

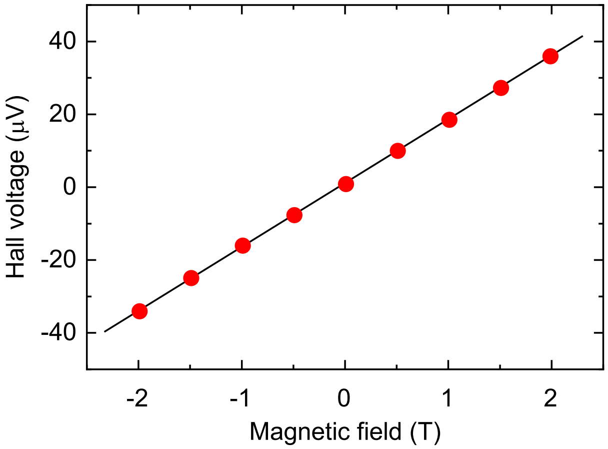

Figure S10: Hall voltage of CaCuP film versus applied magnetic field at constant driving current at room temperature. The Hall voltage at each magnetic field is determined as the average of four measurements (changing the sign of the driving current for two non-equivalent contact geometries). The linearity of the curve indicates reliable determination of the Hall voltage and, therefore, of the carrier mobility. The positive slope of the plot indicates that holes are majority charge carriers in CaCuP.

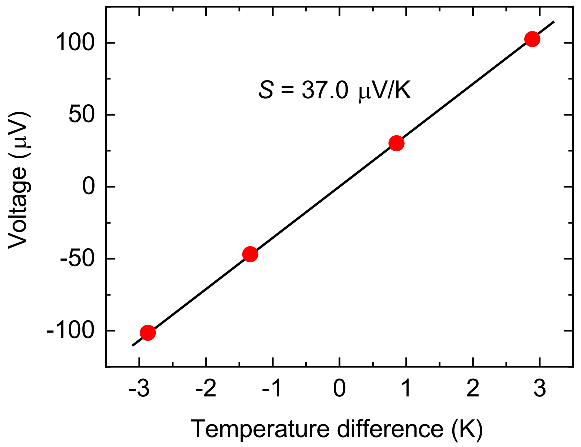

Figure S11: Thermovoltage measurements on a near-stoichiometric CaCuP film at room temperature using a custom-built setup. Two corners of the film are contacted with In wire and one contact is heated to create a temperature gradient. The temperature difference and the voltage between the two contacts are measured after temperature stabilisation. The CaCuP Seebeck coefficient S is extracted as the slope of the thermovoltage versus temperature difference curve, after adding the known Seebeck coefficient of In. The positive sign of S confirms that holes are majority charge carriers in CaCuP. AMSET calculations give a room temperature Seebeck coefficient $67.4\,\mu V\,K^{-1}$, in reasonable agreement considering the differences in conductivity, mobility, etc.

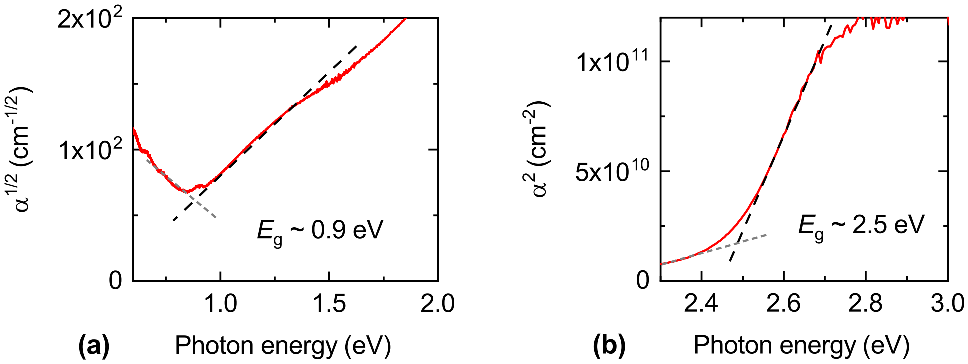

Figure S12: Plots for extrapolating the indirect (a) and direct (b) band gaps assuming a crystalline semiconductor with parabolic density of states at the band edges.[29] α is the absorption coefficient extracted from transmission and reflection measurements (including diffuse components) and shown in the main text.

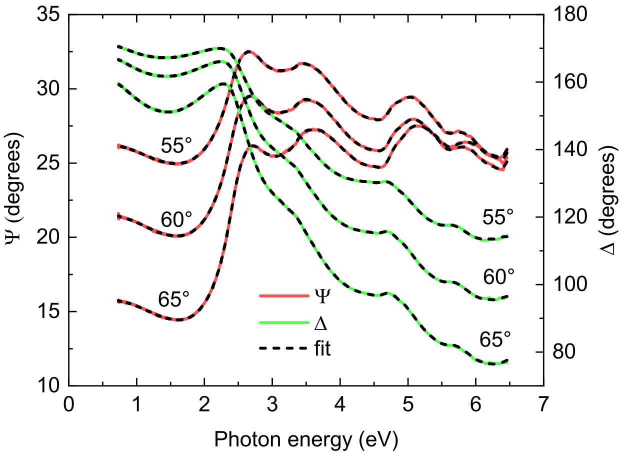

Figure S13: Ellipsometry spectra measured on a thin (61 nm) near-stoichiometric CaCuP film on Si. Ψ and Δ spectra are measured at angles of incidence of 55°, 60° and 65° and fitted simultaneously.

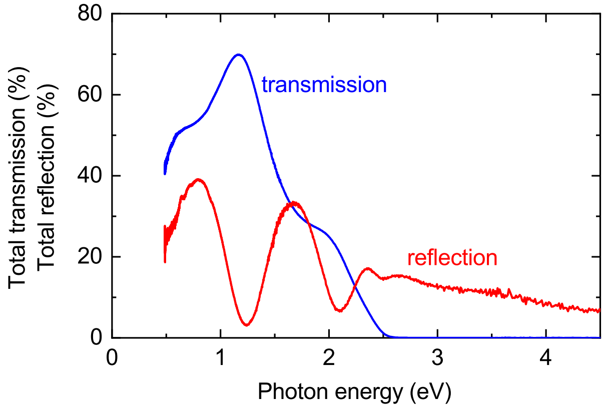

Figure S14: Transmission and reflection measured on a CaCuP film (Film C in XRD Figure in main text). An integrating sphere was used to capture the direct and diffuse components of both transmission and reflection spectra.

References

(1) Talley, K. R.; Bauers, S. R.; Melamed, C. L.; Papac, M. C.; Heinselman, K. N.; Khan, I.; Roberts, D. M.; Jacobson, V.; Mis, A.; Brennecka, G. L.; Perkins, J. D.; Zakutayev, A. COMBIgor: Data-Analysis Package for Combinatorial Materials Science. *ACS Combinatorial Science* **2019**, *21*, 537–547.

(2) Barradas, N. P.; Arstila, K.; Battistig, G.; Bianconi, M.; Dytlewski, N.; Jeynes, C.; Kótai, E.; Lulli, G.; Mayer, M.; Rauhala, E.; Szilágyi, E.; Thompson, M. Summary of "IAEA intercomparison of IBA software". *Nuclear Instruments and Methods in Physics Research, Section B: Beam Interactions with Materials and Atoms* **2008**, *266*, 1338–1342.

(3) Crovetto, A.; Cazzaniga, A.; Ettlinger, R. B.; Schou, J.; Hansen, O. Optical properties and surface characterization of pulsed laser-deposited Cu_2ZnSnS_4 by spectroscopic ellipsometry. *Thin Solid Films* **2015**, *582*, 203–207.

(4) Crovetto, A.; Cazzaniga, A.; Ettlinger, R. B.; Schou, J.; Hansen, O. Large process-dependent variations in band alignment and interface band gaps of Cu_2ZnSnS_4/CdS solar cells. *Solar Energy Materials and Solar Cells* **2018**, *187*, 233–240.

(5) Lany, S.; Zunger, A. Assessment of correction methods for the band-gap problem and for finite-size effects in supercell defect calculations: Case studies for ZnO and GaAs. *Physical Review B* **2008**, *78*, 235104.

(6) Makov, G.; Payne, M. C. Periodic boundary conditions in ab initio calculations. *Physical Review B* **1995**, *51*, 4014–4022.

(7) Lany, S.; Zunger, A. Accurate prediction of defect properties in density functional supercell calculations. *Modelling and Simulation in Materials Science and Engineering* **2009**, *17*, 084002.

(8) Murphy, S. T.; Hine, N. D. M. Anisotropic charge screening and supercell size convergence of defect formation energies. *Physical Review B* **2013**, *87*, 094111.

(9) Jain, A.; Ong, S. P.; Hautier, G.; Chen, W.; Richards, W. D.; Dacek, S.; Cholia, S.; Gunter, D.; Skinner, D.; Ceder, G.; Persson, K. A. Commentary: The Materials Project: A materials genome approach to accelerating materials innovation. *APL Materials* **2013**, *1*, 011002.

(10) Buckeridge, J.; Scanlon, D.; Walsh, A.; Catlow, C. Automated procedure to determine the thermodynamic stability of a material and the range of chemical potentials necessary for its formation relative to competing phases and compounds. *Computer Physics Communications* **2014**, *185*, 330 – 338.

(11) Kunc, K.; Martin, R. M. Ab initio force constants of GaAs: A new approach to calculation of phonons and dielectric properties. *Physical Review Letters* **1982**, *48*, 406–409.

(12) Lloyd-Williams, J. H.; Monserrat, B. Lattice dynamics and electron-phonon coupling calculations using nondiagonal supercells. *Physical Review B* **2015**, *92*, 184301.

(13) Ganose, A. M.; Park, J.; Faghaninia, A.; Woods-Robinson, R.; Persson, K. A.; Jain, A. Efficient calculation of carrier scattering rates from first principles. *Nature Communications* **2021**, *12*, 2222.

(14) Williams, F. Theoretical low temperature spectra of the thallium activated potassium chloride phosphor. *Physical Review* **1951**, *82*, 281–282.

(15) Lax, M. The Franck-Condon principle and its application to crystals. *Journal of Chemical Physics* **1952**, *20*, 1752–1760.

(16) Monserrat, B. Vibrational averages along thermal lines. *Physical Review B* **2016**, *93*, 014302.

(17) Monserrat, B.; Dreyer, C. E.; Rabe, K. M. Phonon-assisted optical absorption in $BaSnO_3$ from first principles. *Physical Review B* **2018**, *97*, 104310.

(18) Morris, A. J.; Monserrat, B. Optical absorption driven by dynamical symmetry breaking in indium oxide. *Physical Review B* **2018**, *98*, 161203.

(19) Bravić, I.; Monserrat, B. Finite temperature optoelectronic properties of BAs from first principles. *Physical Review Materials* **2019**, *3*, 065402.

(20) Brlec, K.; Davies, D. W.; Scanlon, D. O. Surfaxe: Systematic surface calculations. *Journal of Open Source Software* **2021**, *6*, 3171.

(21) Walsh, A.; Butler, K. T. Prediction of Electron Energies in Metal Oxides. *Accounts of Chemical Research* **2013**, *47*, 364–372.

(22) Burton, L. A.; Walsh, A. Band alignment in SnS thin-film solar cells: Possible origin of the low conversion efficiency. *Applied Physics Letters* **2013**, *102*, 132111.

(23) Wei, S.-H.; Zunger, A. Calculated natural band offsets of all II–VI and III–V semiconductors: Chemical trends and the role of cation d orbitals. *Applied Physics Letters* **1998**, *72*, 2011–2013.

(24) Ganose, A. M.; Savory, C. N.; Scanlon, D. O. Electronic and defect properties of $(CH_3NH_3)_2Pb(SCN)_2I_2$ analogues for photovoltaic applications. *Journal of Materials Chemistry A* **2017**, *5*, 7845–7853.

(25) Einhorn, M.; Williamson, B. A. D.; Scanlon, D. O. Computational prediction of the thermoelectric performance of LaZnOPn (Pn = P, As). *Journal of Materials Chemistry A* **2020**, *8*, 7914–7924.

(26) Williamson, B. A. D.; Buckeridge, J.; Brown, J.; Ansbro, S.; Palgrave, R. G.; Scanlon, D. O. Engineering Valence Band Dispersion for High Mobility p-Type Semiconductors. *Chemistry of Materials* **2017**, *29*, 2402–2413.

(27) Mewis, A. ABX-Verbindungen mit Ni_2In-Struktur Darstellung und Struktur der Verbindungen CaCuP(As), SrCuP(As), SrAgP(As) und EuCuAs / ABX Compounds with the Structure Ni_2In Preparation and Crystal Structure of CaCuP(As), SrCuP(As), SrAgP(As), and EuCuAs. *Zeitschrift für Naturforschung B* **1978**, *33*, 983–986.

(28) Ganose, A. M.; Jackson, A. J.; Scanlon, D. O. sumo: Command-line tools for plotting and analysis of periodic ab initio calculations. *Journal of Open Source Software* **2018**, *3*, 717.

(29) Yu, P. Y.; Cardona, M. *Fundamentals of Semiconductors*; Graduate Texts in Physics; Springer Berlin Heidelberg: Berlin, Heidelberg, 2010.
